# Nosocomial Outbreak of New Delhi Metallo-β-Lactamase-1-Producing Gram-Negative Bacteria in South Africa: A Case-Control Study

**DOI:** 10.1371/journal.pone.0123337

**Published:** 2015-04-24

**Authors:** Pieter de Jager, Tobias Chirwa, Shan Naidoo, Olga Perovic, Juno Thomas

**Affiliations:** 1 Epidemiology and Surveillance Unit, National Institute for Occupational Health, National Health Laboratory Service, Johannesburg, South Africa; 2 Department of Community Health, School of Public Health, Faculty of Health Sciences, University of the Witwatersrand, Johannesburg, South Africa; 3 Department of Epidemiology and Biostatistics, School of Public Health, Faculty of Health Sciences, University of the Witwatersrand, Johannesburg, South Africa; 4 Centre for Opportunistic, Tropical and Hospital Infections, National Institute for Communicable Diseases, National Health Laboratory Service, Johannesburg, South Africa; 5 Department of Clinical Microbiology and Infectious Diseases, School of Pathology, Faculty of Health Science, University of Witwatersrand, Johannesburg, South Africa; 6 Outbreak Response Unit, National Institute for Communicable Diseases, National Health Laboratory Service, Johannesburg, South Africa; Alfa Institute of Biomedical Sciences (AIBS), GREECE

## Abstract

**Objective:**

New Delhi metallo-β-lactamase (NDM)-producing Gram-negative bacteria have spread globally and pose a significant public health threat. There is a need to better define risk factors and outcomes of NDM-1 clinical infection. We assessed risk factors for nosocomial infection with NDM-1-producers and associated in-hospital mortality.

**Methods:**

A matched case-control study was conducted during a nosocomial outbreak of NDM-1-producers in an adult intensive care unit (ICU) in South Africa. All patients from whom NDM-1-producers were identified were considered (n=105). Cases included patients admitted during the study period in whom NDM-1 producing Gram-negative bacteria were isolated from clinical specimens collected ≥48 hours after admission, and where surveillance definitions for healthcare-associated infections were met. Controls were matched for age, sex, date of hospital admission and intensive-care admission. Conditional logistic regression was used to identify risk factors for NDM-1 clinical infection and associated in-hospital mortality.

**Findings:**

38 cases and 68 controls were included. *Klebsiella pneumoniae* was the most common NDM-1-producer (28/38, 74%). Cases had longer mean hospital stays (44.0 vs. 13.3 days; *P* < 0.001) and ICU stays (32.5 vs. 8.3 days; *P* < 0.001). Adjusting for co-morbid disease, the in-hospital mortality of cases was significantly higher than controls (55.3% vs. 14.7%; AOR, 11.29; *P* < 0.001). Higher Charlson co-morbidity index score (5.2 vs. 4.1; AOR, 1.59; *P* = 0.005), mechanical ventilation days (7.47 vs. 0.94 days; AOR, 1.32; *P* = 0.003) and piperacillin/tazobactam exposure (11.03 vs. 1.05 doses; AOR, 1.08; *P* = 0.013) were identified as risk factors on multivariate analysis. Cases had a significantly higher likelihood of in-hospital mortality when the NDM-1-producer was *Klebsiella pneumoniae* (AOR, 16.57; *P* = 0.007), or when they had a bloodstream infection (AOR, 8.84; *P* = 0.041).

**Conclusion:**

NDM-1 infection is associated with significant in-hospital mortality. Risk factors for hospital-associated infection include the presence of co-morbid disease, mechanical ventilation and piperacillin/tazobactam exposure.

## Introduction

Resistance to β-lactams is a long recognised problem in Gram-negative bacteria[1] and with the introduction of new classes of β-lactams, novel β-lactamases have emerged.[1,2] Carbapenem resistance has become a growing problem over the last decade with the emergence of readily transferable plasmid mediated carbapenem-hydrolysing β-lactamases. [3,4] These carbapenemases constitute a heterogeneous and versatile group of enzymes hydrolysing β-lactams and also exhibit resistance to β-lactamase inhibitors, making them exceedingly difficult to treat.[4,5]

In 2008 a novel metallo-β-lactamase designated New Delhi metallo-β-lactamase (NDM-1) was identified in a Swedish patient returning from India.[6] The first case of NDM-1 in South Africa was identified in September 2011.[7] The *bla*_NDM-1_ gene is plasmid mediated and associated with numerous other resistance determinants conferring resistance to β-lactams, fluoroquinolones and aminoglycosides resulting in significant treatment option limitations.[4,8] Sensitivity to tigecycline and polymyxins are typically reserved although the efficacy of these treatment options have not been established and drug toxicity, particularly with colistin, poses further clinical challenges.[9] Compared to other carbapenemase types, NDM-1 displays a broader spectrum of antimicrobial resistance and its global spread has been singularly rapid; notably, it has been detected in diverse species and genera of Gram-negative bacteria.[10,11] NDM-1-producers have been documented on every continent except Antarctica,[12–14] with increasing reports of transmission and acquisition of NDM-1-producers both in healthcare facilities and in the community.[15,16]

With limited treatment options available, slowing and preventing the spread of *bla*_NDM-1_ through an understanding of risk factors for its acquisition is essential. However, there is a paucity of published epidemiological studies reporting on risk factors for NDM-1 clinical infection. In order to evaluate risk factors for NDM-1 clinical infection and associated in-hospital mortality, we conducted a matched case-control study during a prolonged outbreak of NDM-1 producing Gram-negative bacteria in a South African hospital. We hypothesised that exposure to antibiotics and medical devices would be risk factors for NDM-1 clinical infection and would be associated with greater in-hospital mortality.

## Methods

### Ethics statement

Study participant age ranged from 20 years to 90 years, with a mean age of 61.3 and a median age of 64. Verbal informed consent was obtained from all patients or their next of kin prior to conducting telephonic interviews which collected information on past hospitalization/chronic care admission and travel history. Verbal consent was obtained as this was a retrospective study and patients had subsequently relocated to various parts of the country. Consent was captured on a consent form by the researchers. Consent to review clinical records were obtained from the hospital and all patient data were anonymized and de-linked from unique identifiers prior to analysis. Ethics approval for this study, including the consent procedure, was obtained from the Human Research Ethics Committee (Medical) at the University of the Witwatersrand, Johannesburg. (M130248)

### Study setting and laboratory methods

The outbreak occurred across three private hospitals in South Africa with strong referral links. This study was confined to the hospital where the majority of cases (90/105, 86%) were detected, and all cases and controls were from the adult ICU. The hospital is located in the greater Johannesburg area, has a total of 322 beds including a 37-bed ICU, and offers tertiary-level specialist care.

In early August 2011 *Klebsiella pneumoniae* isolated from an 86-year-old male admitted following a hip fracture was found to harbour *bla*_NDM-1_. In response to this, the first case of NDM-1 both in the hospital and the country, a rectal screening programme was instituted to identify patients colonised with NDM-1-producers, with screening criteria revisions throughout the course of the outbreak. The method of screening employed by all diagnostic laboratories throughout the outbreak was direct real-time polymerase chain reaction (RT-PCR) testing for *bla*_NDM-1_ on dry rectal swabs. Clinical isolates demonstrating phenotypic resistance to carbapenems were further tested for *bla*_NDM-1_ using RT-PCR. The LightCycler 480 II (Roche Applied Science) instrument was used for the RT-PCR assay. The *bla*_NDM-1_ gene was amplified by a real time polymerase chain reaction (PCR) using the LightCycler 480 Probes Master kit (Roche Diagnostics, IN, USA) and the LightMix Modular NDM (ESBL) kit (Roche Diagnostics, IN, USA). The positive control was provided with the LightMix Modular kits and sterile water was used as a negative control. An internal control, the LightMix Modular PhHV Internal Control kit (Roche Diagnostics, IN, USA) was also included in the run.

All microbiological testing was conducted in routine private diagnostic laboratories servicing the private healthcare sector. Thirteen NDM-positive isolates (seven case isolates and six epidemiologically linked environmental specimens collected in the adult ICU) were subjected to DNA fingerprinting by macro-restriction analysis on pulsed-field gel electrophoresis (PFGE) at the Infection Control Services Laboratory, National Health Laboratory Services. PFGE was performed as described previously.[17]

### Cases and Controls

**Fig 1:**
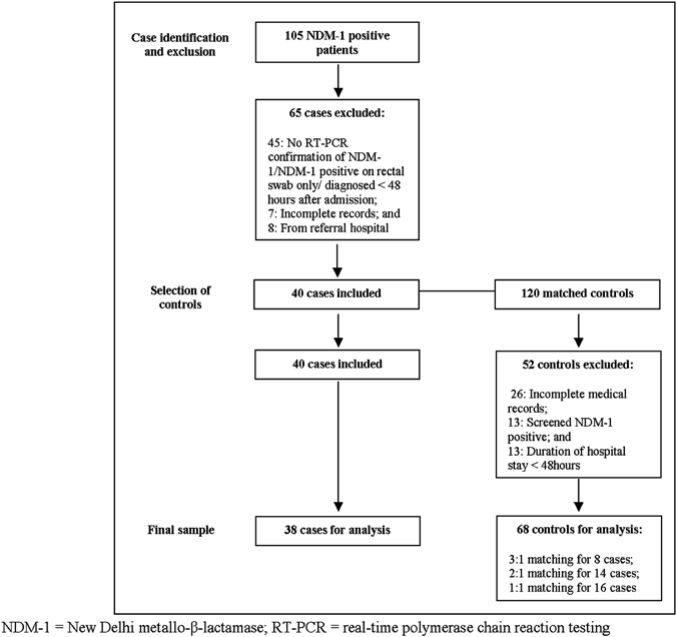
Study design and selection of cases and controls. NDM-1 = New Delhi metallo-β-lactamase; RT-PCR = real-time polymerase chain reaction testing.

The study design is summarized in Fig 1.

All patients admitted between 1 July 2011 and 31 October 2012 were eligible for inclusion. We included cases where *bla*_NDM-1_ was detected on an isolate from a specimen collected at least 48 hours after admission and the infection was categorised as a healthcare-associated infection as per the Centers for Disease Control and Prevention/National Healthcare Safety Network definitions.[18] Potential cases were excluded if *bla*_NDM-1_ was detected on rectal screening alone, where rectal screening was positive within the first 48 hours of admission, or where clinical records were incomplete. After exclusion of cases not fulfilling the inclusion criteria, 40 cases remained and three controls were matched to each case for sex (male/female), age (+/- 5 years), date of hospital admission (+/- 14 days) and ICU admission (yes/no). Where more than three eligible controls were identified on the hospital’s electronic database, three controls were randomly selected. Controls were excluded if they had *bla*_NDM-1_ detected on any sample during the hospitalisation period, if patient records were incomplete or missing, or if the patient was admitted for less than 48 hours.

No controls could be found meeting the matching criteria for two cases and for three cases only two matching controls could be identified. Another 52 controls were excluded for missing/incomplete medical records (n = 26), record of screening NDM-1 positive on dry rectal swab (n = 13) or being admitted for less than 48 hours (n = 13). The final sample consisted of 38 cases and 68 controls.

### Data collection

Clinical data were collected for cases and controls from clinical records, laboratory results and hospital billing data. Exposure to antibiotics (carbapenems, aminoglycosides, fluoroquinolones, third- and fourth-generation cephalosporins, and piperacillin/tazobactam) and corticosteroids were recorded as total number of doses received. Exposure to medical devices (central venous line and indwelling urinary catheter) as well as selected medical interventions (haemodialysis, mechanical ventilation and parenteral nutrition) was recorded as total number of days exposed. Patients who underwent laparotomy or thoracotomy were grouped and compared to patients who received other (mainly orthopaedic) or no surgery. Co-morbid disease and severity of illness on admission was measured by calculating Charlson co-morbidity index and Mortality Probability Model III (MPM III) scores respectively.[19,20] Exposure data for cases were collected from the date of admission until the date of collection of the first sample yielding an NDM-1-producing isolate (time at risk). For controls, exposure data were collected from the date of admission until the date of discharge or death (time at risk). Beyond the time at risk, total length of hospital and total length of intensive care unit stay were also collected.

Data on past hospital or long-term care facility admission and international travel history in the year leading up to the admission of interest were collected through telephonic interviews for both cases and controls. All data were collected between November 2012 and November 2013 by trained professional nurses and medical doctors.

### Statistical analysis

We evaluated risk factors associated with case status and compared in-hospital mortality between cases and controls. Except for MPM-III scores, where its calculation would have been invalid, there was no missing clinical data in the final sample used for analysis. Where past admission, travel history or MPM-III scores were missing, observations were excluded from the analysis.

Data were entered into Epi-Info version 7 and exported to Excel 2007 where it was inspected for errors before being imported to Stata Version 12 for statistical analysis. Continuous variables such as length of hospital stay, MPM-III and Charlson scores, are described through the reporting of means and standard deviations. Two sided t-test for two groups (cases and controls) was used to compare means of continuous variables with normal distributions. Where data were not normally distributed Mann-Whitney U test was used. For differences in proportions such as previous hospitalisation or travel history, Mantel–Haenszel Chi square test was used. Bivariate conditional logistic regression analysis was undertaken to calculate crude odds ratio’s for exposure to medical devises and interventions, antibiotics and duration of stay. Stepwise conditional logistic regression was conducted to identify factors associated with case status. All exposure variables with a *P* < 0.20 at the univariate level were considered in the final multiple regression model. Significance was taken at a level of 0.05. Conditional logistic regression was further undertaken to calculate the odds of in-hospital mortality for cases and controls as well as for different sites of infection and clinical isolates. Adjusted odds ratios were calculated using multivariable conditional logistic regression.

## Results

The most common NDM-1-producing isolate among the 38 cases was *Klebsiella pneumoniae* (28/38, 74%) followed by *Enterobacter cloacae* (5/38, 13%), *Klebsiella oxytoca* (2/38, 5%), *Serratia marcescens* (2/38, 5%) and *Citrobacter amalonaticus* (1/38, 3%). The most common clinical specimen types yielding NDM-1 were sputum (16/38, 42%), blood (12/38, 32%) and urine (5/38, 13%) followed by pus (2/38, 5%), broncho-alveolar lavage (2/38, 5%) and pleural fluid (1/38, 3%).

PFGE showed two closely related clusters: cluster A comprised three case isolates and six environmental isolates, whilst cluster B comprised three case isolates. Given the protracted course of the outbreak, this suggests that these isolates are all related.[21]

Cases had a longer mean total length of hospital stay (44.0 vs 13.3 days, *P* < 0.001) and longer mean durations of time at risk, particularly mean ICU time at risk (18.9 vs 8.3 days, *P* <0.001) than controls (Table 1). Charlson co-morbidity index scores were, on average, significantly higher in cases than controls (5.2 vs 4.1, P = 0.032).

**Table 1:** Duration of stay, time at risk and co-morbid status for cases and controls.

Variable	Cases (n = 38) Mean (SD)	Controls(n = 68) Mean (SD)	*p*-value
Time at risk (total, days)	22.2 (±15.8)	13.3 (±9.5)	**0.004**
Time at risk (intensive care, days)	18.9 (±13.7)	8.3 (±7.2)	**<0.001**
Total length of stay (days)	44.0 (±28.2)	13.3 (±9.5)	**<0.001**
Total length of ICU stay (days)	32.5 (±27.0)	8.3 (±7.2)	**<0.001**
MPM III Score (%)	11.5 (±7.1)	8.3 (±6.8)	0.072
Age Adjusted Charlson Score	5.2 (±3.1)	4.1 (±2.2)	**0.032**

SD = standard deviation; time at risk: from admission to discharge/death (controls) or NDM-1 diagnosis (cases); MPM-III = Mortality Probability Model III; total length of stay: time from admission to discharge/death; *p*-values calculated using Mann-Whitney U test.

### Risk factors associated with case status

Cases had significantly higher odds of having been hospitalised or admitted to a long-term care facility in the previous year (OR 6.83; 95% CI 2.32–20.16) or being transferred from a referral hospital (OR 4.98; 95% CI 1.56–15.93) compared to controls (Table 2). No association was found between travel history and case status. Although total time at risk was not associated with case status, an ICU stay of longer than seven days was associated with a significant risk of infection with NDM-1-producers (OR 4.82; 95% CI 1.80–12.91). Exposure to any antibiotics (carbapenem, fluoroquinolone, aminoglycoside, third- or fourth-generation cephalosporins, or piperacillin/tazobactam) was also significantly associated with case status (OR 4.77; 95% CI 1.38–16.48). No association between HIV status or surgery (laparotomy or thoracotomy) and infection with NDM-1-producers was found.

**Table 2:** Univariate analysis of pre-hospital factors, HIV status, time at risk, surgery and antibiotic exposure among cases and controls.

Exposure Variable	Case patient (n = 38)with exposure		Control patient (n = 68) with exposure		Unadjusted OR(95%CI)	*p*-value
	Number	%	Number	%		
Previous Hospitalization/Chronic care						
No	10	29	40	83	1	
Yes	24	71	8	17	6.83 (2.32–20.16)	**<0.001**
Travel outside South Africa						
No	30	94	47	98	1	
Yes	2	6	1	2	3.24 (0.29–36.63)	0.343
Transfer from referral hospital						
No	23	61	60	88	1	
Yes	15	39	8	12	4.98 (1.56–15.93)	**0.007**
HIV Status						
HIV negative	34	89	63	93	1	
HIV positive	4	11	5	7	1.53 (0.29–8.11)	0.615
Time at risk (total)						
≤ 14 days	17	45	44	65	1	
> 14 days	21	55	24	35	2.12 (0.97–4.62)	0.059
Time at risk (intensive care)						
1–7 days	9	24	40	59	1	
>7 days	29	76	28	41	4.82 (1.80–12.91)	**0.002**
Surgery*						
No	14	37	33	49	1	
Yes	24	63	35	51	1.60 (0.72–3.56)	0.254
Exposure to antibiotics**						
No	5	13	27	40	1	
Yes	33	87	41	60	4.77 (1.38–16.48)	**0.014**

*Refers to laparotomy or thoracotomy;

**Refers to receiving any dose or either a carbapenem or fluoroquinolone or aminoglycoside or third/fourth generation cephalosporin or piperacillin/tazobactam;

OR = odds ratio

On univariate analysis exposure to aminoglycosides, piperacillin/tazobactam and corticosteroids were significantly associated with case status (Table 3). Each additional dose of piperacillin/tazobactam or a corticosteroid was associated with a 5% increase in risk of developing infection with a NDM-1-producer, while each additional dose of an aminoglycoside was associated with a 3% increase in risk. Although exposure to fluoroquinolones, carbapenems and third-/fourth-generation cephalosporins were associated with an increased risk of case status, none of these showed statistical significance at the 5% level. Each additional day of exposure to a central venous line or indwelling urinary catheter was associated with an 8% and 7% increased risk of case status on univariate analysis respectively. Selected medical interventions were significantly associated with NDM-1-producer infection, with a 16% and 27% increased risk for each additional day of haemodialysis and mechanical ventilation respectively. As summarised in Table 4, multivariate analysis showed mechanical ventilation and exposure to piperacillin/tazobactam to be significantly associated with case status.

**Table 3:** Univariate analysis of exposure to antibiotics (aminoglycosides, fluoroquinolones, carbapenems, third/fourth generation cephalosporins and piperacillin/tazobactam), corticosteroids, invasive medical devices and selected medical interventions among cases and controls.

Exposure Variable	Case patient (n = 38) with exposure	Control patient (n = 68) with exposure	Crude OR (95%CI)	*p*-value
	mean (SD)	mean (SD)		
Aminoglycosides (dose, any)	10.42 (±22.53)	2.43 (±10.23)	1.03 (1.00–1.06)	**0.043**
Gentamycin	0.97 (±5.35)	0.25 (±1.74)	1.07 (0.93–1.23)	0.320
Amikacin	7.29 (±18.79)	2.17 (±10.05)	1.02 (0.99–1.06)	0.125
Tobramycin	2.16 (±13.30)	0 (±0)	-	-
Fluoroquinolone (dose, any)	1.53 (±3.75)	0.91(±2.76)	1.09 (0.96–1.24)	0.162
Ciprofloxacin	0.71 (±3.02)	0.16 (±1.00)	1.19 (0.90–1.57)	0.234
Levofloxacin	0.66 (±2.33)	0.49 (±2.32)	1.07 (0.91–1.26)	0.429
Moxifloxacin	0.15 (±0.97)	0.26 (±1.32)	0.96 (0.67–1.38)	0.830
Carbapenem (dose, any)	16.08(±29.93)	5.59(±11.97)	1.02 (1.00–1.05)	0.062
Doripenem	6.16 (±18.43)	0.15(±1.21)	1.18 (0.96–1.46)	0.117
Ertapenem	1.39 (±4.03)	1.22 (±3.56)	0.99 (0.88–1.12)	0.930
Meropenem	8.52 (±16.74)	4.22 (±11.17)	1.02 (0.99–1.05)	0.175
Cephalosporin (dose, any)	2.5 (±7.07)	2.19 (±6.0)	1.00 (0.94–1.06)	0.992
Cefepime	1.68 (±6.43)	0.51 (±3.07)	1.06 (0.96–1.16)	0.240
Ceftriaxone	0.82 (±2.82)	1.67 (±4.93)	0.93 (0.83–1.04)	0.201
Pip-tazobactam (dose)	11.03 (±12.10)	6.17 (±10.31)	1.05 (1.02–1.10)	**0.015**
Steroids (dose, any)	23.5 (±23.93)	7.22 (±12.96)	1.05 (1.02–1.09)	**0.003**
Invasive Medical Devices				
Central venous line (days)	15.42 (±14.66)	6.51 (±6.71)	1.08 (1.03–1.13)	**0.003**
Urinary catheter (days)	18.61 (±15.92)	7.35 (±7.93)	1.07 (1.03–1.12)	**0.001**
Medical Interventions				
Mechanical Ventilation (days)	7.47 (±8.55)	0.94 (±2.34)	1.27 (1.10–1.48)	**0.001**
Parental Nutrition (days)	2.53 (±3.40)	1.40 (±3.83)	1.07 (0.96–1.20)	0.217
Haemodialysis (days)	6.03 (±14.3)	0.68 (±2.74)	1.16 (1.01–1.33)	**0.030**

SD = standard deviation; OR = odds ratio.

**Table 4:** Multiple conditional logistic regression analysis for factors associated with case status.

Exposure Variable	Adjusted OR (95% CI)*	*p*-value
Charlson co-morbidity index score	1.59 (1.15–2.18)	**0.005**
Mechanical Ventilation (days)	1.32 (1.10–1.59)	**0.003**
Piperacillin/tazobactam (dose)	1.08 (1.02–1.15)	**0.013**

* Adjusted for Charlson co-morbidity index score, mechanical ventilation and piperacillin/tazobactam; OR = odds ratio.

Of the 68 controls 10 died in hospital (14.7%), while 21 of the 38 cases died in hospital (55.3%);this translates to an attributable mortality of 47.5% (Table 5). After adjusting for co-morbid disease, case status was associated with an eleven-fold higher risk of in-hospital mortality (AOR 11.29; 95% CI 2.57–49.60) compared to controls. Cases with bloodstream infections due to NDM-1-producers (AOR 8.84; 95% CI 1.09–71.55), or where the organism harbouring the *bla*_NDM-1_ was *Klebsiella pneumoniae* (AOR 16.57; 95% CI 2.12–129.6) had a significantly higher likelihood of in-hospital mortality.

**Table 5:** Risk factors associated with in-hospital mortality.

Variable	Death (n = 31) n(%)	Unadjusted OR (95% CI)	*p*-value	Adjusted* OR (95% CI)	*p*-value
Case—Control					
Control	10 (32)	1		1	
Case	21 (68)	12.81 (2.94–55.82)	**0.001**	11.29 (2.57–49.60)	**0.001**
Site of Infection					
None	10 (32)	1		1	
Pneumonia	11 (36)	5.5e (-)	0.994	3.54e(-)	0.993
Blood stream infection	8 (26)	9.03 (1.10–74.21)	**0.041**	8.84 (1.09–71.55)	**0.041**
Other	2 (6)	4.37 (0.37–51.24)	0.240	3.51 (0.28–44.71)	0.333
Isolate					
None	10 (32)	1		1	
*Klebsiella pneumoniae*	16 (52)	19.30 (2.50–148.83)	**0.005**	16.57 (2.12–129.6)	**0.007**
Other GNB	5 (16)	6.36 (0.72–56.51)	0.097	6.08 (0.69–53.90)	0.105

*Adjusted for Charlson co-morbidity index; OR = odds ratio; GNB = Gram-negative bacteria.

## Discussion

To our knowledge this is the largest epidemiological study investigating risk factors and in-hospital mortality associated with clinical infection during an outbreak of NDM-1-producers, and adds evidence to support rational preventive and control measures. We found that higher Charlson co-morbidity scores, mechanical ventilation and piperacillin/tazobactam exposure were independently associated with infection with NDM-1-producers. Secondly, in-hospital mortality was found to be significantly higher in patients with clinical infection due to NDM-1-producers. Molecular strain typing of NDM-1-producing *Klebsiella pneumoniae* isolates from cases and the environment supported the hypothesis of horizontal transmission occurring in the ICU.

We identified three previously published papers reporting on risk factors for infection with NDM-1-producers. The first was a review of reported cases (n = 77) across the European Union which found travel to India, Pakistan or the Balkans to be associated with NDM-1 acquisition.[22] The second study was a case series (n = 5) of a nosocomial outbreak of carbapenem resistant enterobacteriaceae harbouring *bla*_NDM-1_ in Canada.[23] The third study by Lowe *et al*.[24] investigated nosocomial transmission of NDM-1 to seven patients from two index cases and found exposure to fluoroquinolones, trimethoprim-sulfamethoxazole and carbapenems to be possible risk factors for NDM-1 acquisition.[24] We found exposure to both carbapenems and fluoroquinolones to be associated, albeit not significantly, with subsequent infection due to a NDM-1-producer. We did not assess trimethoprim-sulfamethoxazole exposure in our study as it was not commonly prescribed in the setting of this outbreak. Our analysis shows aminoglycoside and piperacillin/tazobactam exposure to be significantly associated with case status at the univariate level, and piperacillin/tazobactam was found to be an associated with clinical infection with NDM-1-producers after adjusting for co-morbid disease.

Our findings that an increased duration of exposure to central venous lines, urinary catheters, mechanical ventilation and haemodialysis were associated with an increased risk of infection with NDM-1-producers are consistent with risk factors for the acquisition of carbapenemase-producers identified by previous investigators. Medical devices such as urinary catheters[25,26] and central venous lines[25–27] as well as interventions such as mechanical ventilation[25,27,28] and haemodialysis[25] are well-established risk factors. These risk factors have also been found consistent in the acquisition of IMP-type metallo-β-lactamase producing Gram-negatives.[29,30] This is the first study that identifies and quantifies these exposures for NDM-1-producers.

Of the early NDM-1 cases detected in the United States and United Kingdom, many had epidemiological links to India and Pakistan.[4,15] We found no association between international travel and case status. Despite not being able to complete the telephonic interview for all cases (32 completed/38, 84%) or controls (48 completed/68, 71%), we would argue that international travel was not a risk factor for NDM-1 acquisition in the cases linked to this nosocomial outbreak. Of the first five cases identified in the outbreak, none reported any travel history in the year preceding admission, and none of the cases interviewed telephonically reported travel to India, Pakistan or the Balkans, which had been identified as high NDM-1-transmission regions at the time of the outbreak.[4,15] In India, Gram-negative bacteria surveillance isolates collected two years prior to the first identification of NDM-1 has subsequently been shown to harbour *bla*_NDM-1_.[31] Similarly, given the lack of standardised surveillance in South Africa, it is likely that *bla*_NDM-1_ had been present in clinically-relevant bacteria for some time before the index case was identified.

In-hospital mortality for extended-spectrum beta-lactamase producers has been reported at around 37%[32] and amongst patients with carbapenem-resistant *Klebsiella pneumoniae* at between 44% and 48%.[25,28] Crude mortality in patients with bloodstream infections caused by KPC-producing *Klebsiella pneumoniae* is estimated at 53%.[33] Given these reported mortality rates and the limited treatment options available for NDM-1-producers, our finding of a 55.3% crude in-hospital mortality rate was to be expected. However, considering this outbreak occurred in a well-resourced private sector hospital, mortality rates in patients with similar infections cared for in public sector hospitals in South Africa would be expected to be higher due to limited available antibiotics and ICU facilities. This would likely be the case in many under-resourced healthcare facilities worldwide, which further underscores the importance of taking preventive action to reduce transmission of such multidrug-resistant organisms in the hospital setting, thereby preventing nosocomial outbreaks and limiting dissemination into the community.

The hospital undertook a range of interventions to control the outbreak. Patients found to be colonized with NDM-1-producers through the rectal screening program were cohorted and assigned dedicated nursing staff and medical equipment. Healthcare workers and cleaning staff received targeted education about the importance of hand hygiene, and stringent hand washing protocols were instituted throughout the hospital. Hand hygiene practice in the adult ICU was monitored for compliance through closed circuit television. Infrastructural alterations to the adult ICU increased the ICU’s capacity to effectively isolate patients. Weekly meetings were attended by multidisciplinary role-players, including the hospital infection prevention and control practitioners, hospital management staff, medical microbiologists, hospital clinicians, and members of the National Institute for Communicable Diseases’ Outbreak Response Unit. Controlling the outbreak was resource intensive and demanded a concerted effort from all role-players, with critical review of the outbreak situation and re-evaluation of interventional strategies throughout. Although sporadic cases of colonisation or infection with NDM-1-producers continue to be reported, no clusters or epidemiologically-linked cases have been identified since the end of the outbreak.

This study has a number of limitations. Due to the inherent nature of outbreak investigations, there were a limited number of potential cases. All potential cases were reviewed and as many matching controls as were available were included. However, the small sample size limits the study’s power to detect other antimicrobial agents as risk factors for infection with NDM-1-producers. The outbreak was confined to the adult ICU, limiting generalisability to a paediatric population. Missing clinical records and missing data on international travel and previous admissions in the year leading up to the admission of interest reduced our sample size and ability to evaluate pre-hospitalization risk factors. The fluctuating point prevalence of NDM-1-producers and the clinicians’ enhanced diagnostic suspicion of infection with NDM-1-producers as the outbreak evolved may bias findings. We addressed this, however, by matching controls for date of hospital admission.

Given the dearth of new antimicrobials in the drug development pipeline, the burgeoning threat of conquer by virtually untreatable multidrug-resistant organisms of clinical relevance is becoming realised thanks to the emergence and rapid spread of, amongst others, the carbapenemases.[34,35] Through a better understanding of the risk factors and epidemiological characteristics of patients developing clinical infection with NDM-1-producers, infection prevention and control practice and antimicrobial stewardship programs can be tailored to identify vulnerable patients and prioritise areas for risk reduction, both in an outbreak situation and beyond. This study contributes to a growing body of knowledge for action by identifying risk factors for infection with NDM-1-producers, and highlights the ‘bottom line’—such infections exact significant mortality and swift, effective action is needed.
